# ‘Digital Wellbeing’: The Need of the Hour in Today’s Digitalized and Technology Driven World!

**DOI:** 10.7759/cureus.27743

**Published:** 2022-08-07

**Authors:** Nisha M Thomas, Sonali G Choudhari, Abhay M Gaidhane, Zahiruddin Quazi Syed

**Affiliations:** 1 Community Medicine, Datta Meghe Institute of Medical Sciences, Wardha, IND

**Keywords:** digital detox, digital addiction, screen time, health technology, digital wellbeing

## Abstract

The constant contact and usage of technology in today's world have brought about the dire consequences of digital addiction and its effects. This has led to a serious dilemma of management of screentime by an individual. Studies have shown a negative impact of excessive gadget use leading to a decline in performance rates, effect on sleep patterns, and reduction in workplace achievements thereby causing hindrance in unlocking the maximum potential of an individual. This has paved the way for the introduction of a novel concept known as ‘Digital well-being’ for tackling this underlying issue to bring about screen time reduction as well as to establish an ideal work-life balance. Digital well-being enhances the usage of technology itself to combat increased screen time by using restraints and promotes wellness by enabling productive and healthy lifestyles. In a new era where smartphones and technology have begun to dictate our lives, it is necessary to apply restraints and ensure there is a balance of wellness as well as productivity outflow. Digital well-being can be achieved by interventions that should be administered with the use of apps and healthy practices. The use of new-age apps acts as positive reinforcement and helps in providing a restrictive environment as well as maintains the time invested for useful and productive engagements. There is a lot of research yet to be done regarding this topic empirically regarding its success and this review article aims to approach the effectiveness of digital wellbeing and its applications in combating stress and increasing work performance and preventing digital addiction.

## Introduction

Over the past years, there’s been excessive and infiltrating use of smartphones, laptops, and tablets, which have become an integral part of our lives as it keeps us in touch with the external world. Their usage as a means of connectivity and the providence of using it whenever and wherever we want has caused it to be introduced recurrently in our daily life [1]. A dramatic spike has been observed in their usage that has now slowly developed into an unconscious, effortless reach-out for gadgets even when it isn’t mandatory. There has been a major overtaking of gadgets and technology as it has been incorporated into our private and professional lives and has proven to be an aid in achieving a comfortable and easily accessible lifestyle. [2]

It has been found that their design to lure attention has enabled an average person to tap and swipe their phones around 2600 times a day [2] and has caused an individual to spend approximately three to five hours per day [3,4]. It has been studied that there is a risk of addiction in university students all across the globe due to the constant use of gadgets that causes abandonment of work and engagement in unnecessary screen time [5-6]. There has been an observational decline in their academic performance and energy due to lack of sleep which has further added to a stressful lifestyle [6-8]. Studies have established the impact of digital technology usage and teenage well-being to be harmful but to a small proportion [9-10]. Gadgets, having given the leverage and opportunity to users to utilize the power of technology whenever possible, have many downsides and always come with a cost. The constant connection has led to social pressure to constantly be in touch with peers, safety issues due to excessive usage, and an overall decrease in mood and well-being [11]. Due to the negative impact of technology on student health and lower levels of well-being, the need for a novel idea to combat this issue has become a dire need.

## Technical report

Concept of digital well-being 

Digital well-being is an upcoming intervention that can be viewed as using digital technology to ensure one's mental and physical health in an environment overridden by digital abundance. Digital well-being primarily focuses on incorporating and adapting personal tech habits to fulfill essential targets. The central but small steps that can be incorporated into one's life are to promote focus when working or studying and minimize distractions, set reminders to unplug and detox and promote the building of social and family relations for better mental wellness [12]. Digital wellness essentially prioritizes the level of self-control one can assert over their usage of digital devices and focuses on aligning them to achieve long-standing goals. Self-control as the focus of attaining digital well-being is seen to be more effective, and a means to achieve personal and healthy lifestyles [13].

Although individuals have expressed an interest in setting self-limits and restricting the usage of smartphones, adhering to them has proven to be tricky and often cumbersome. There has been the development of many productivity-promoting apps and tools for individuals to set restraints and limits. Still, little research has been done on its effectiveness and the aspects to be considered to ensure its efficiency. Only when there is non-use of apps that cause distractions can there be a shifting of the attention to engage in n productive environment.

Implications of excessive use of digital technology 

The current evidence suggests that typical or balanced digital technology use will not harm adolescents or students. However, excessive or inappropriate use may lead to adverse effects like insufficient sleep [5,9], lack of energy, poor academic achievement [5], altered psychological well-being [6], withdrawal, functional impairment, compulsive behavior [7], physiological stress, mind wandering, attention deficit-hyperactivity disorder-related behavior, nonadaptive/negative thinking styles, decreased life satisfaction [9]. Digital technology use is more likely to affect short-term positive or negative affect than long-term life satisfaction.

Interventions for achieving digital well-being 

The European Commission 2020 announced ‘The European Digital Strategy’ that focuses on digital inclusion incorporating technology in education as one of the priorities in schools and colleges; hence use of technological advancements is inevitable [14]. A study conducted in Riyadh on medical students showed that 51.6% of students use personal digital assistance devices such as laptops and tablets for their studies. Even though they are aware of the ill effects it causes on their health, they continue using the same [4].

Usage of apps

**Figure 1:**
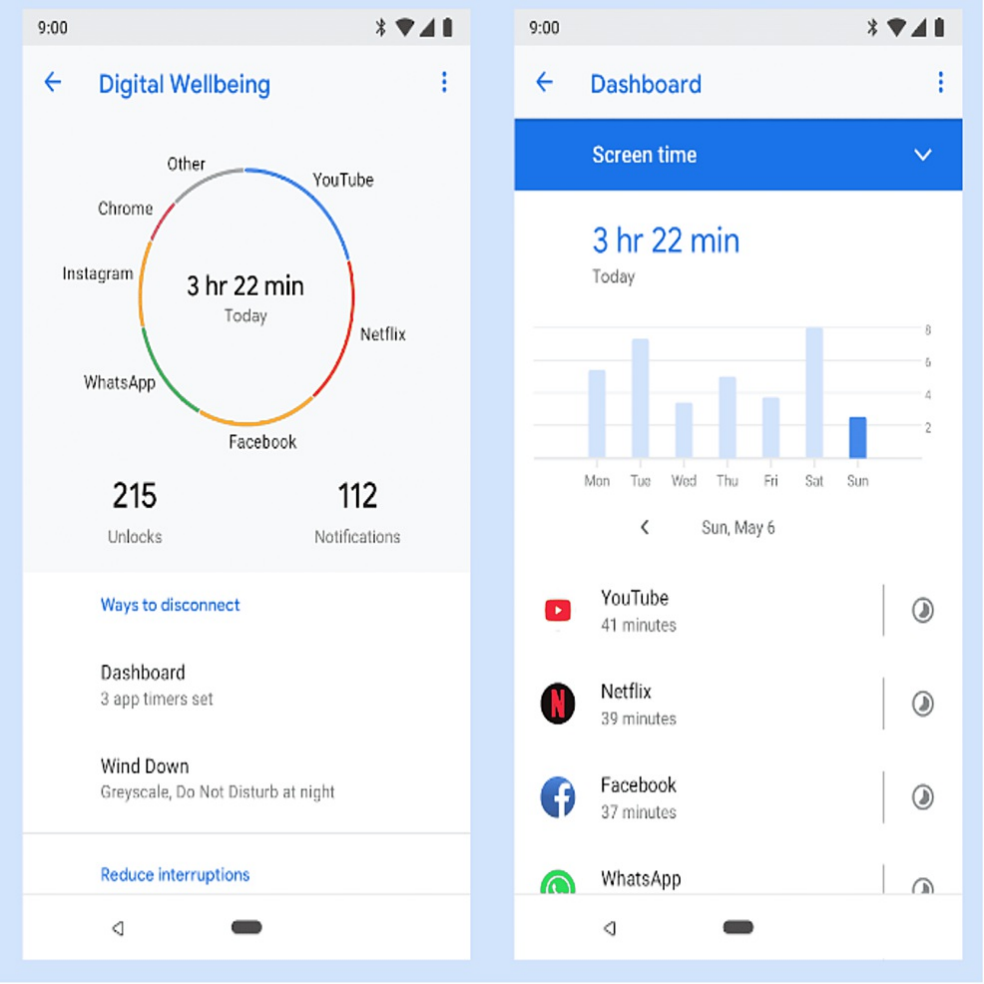
Digital Wellbeing App by Google

**Figure 2:**
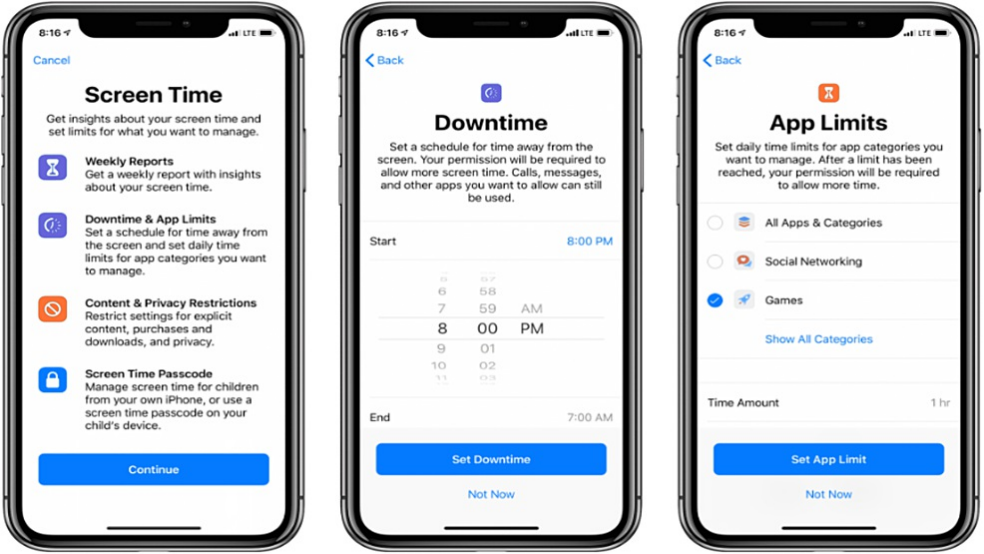
Digital Wellbeing App by IOS

Leading technology companies such as Apple and Google have taken the initiative of incorporating digital well-being tools such as ‘Screentime’ and Digital Wellbeing’ into their operating systems in the promotion of digital wellness by aiding in the monitoring of daily usage and setting limits on distracting apps and ensures concentration and efficiency (Figure 1, 2). Apps such as ‘Forest,’’ Detox’, ’OffTime’, and ‘Moment’ act as positive reinforcement and enable individuals to focus on their goals with visual stimulus and rewards, thereby regaining control of lost screen time [15]. The main focus of such an initiative is to ensure that we use technology for the betterment and not let it take control of our lives.

Research conducted for two weeks on the effectiveness of digital tools with a self-designed app called 'Mytime' revealed that stand-alone interventions have reduced smartphone's non-use of specific apps by 21% and have effectively helped individuals achieve their self-defined goals for a short duration. However, this has been limited as smartphone usage has become a social practice, and often warnings are ignored in a social setting; hence interventions have to be personalized to cater to an individual for them to be effective [11].

NUGU is a group-based app used to self-regulate and restrict smartphone usage. It primarily focuses on taking aid of groups and social support and serves the purpose of helping and motivating each other. There was a positive impact, and this kind of intervention successfully managed distractions and limited usage [16].

Digital detox

Digital detox interventions wherein an individual observes voluntary abstinence from social media and technology have been suggested as a solution to reduce the negative impacts of smartphone use on outcomes like well-being or social relationships. These interventions are a solution to minimize digital addiction [17]. The national day of unplugging observed on 4th-5th March has been followed by many organizations for several years and has dedicated a day to promoting 24 hours detox from technology. Digital detox intervention studies have shown that there has been a significant reduction in stress, improved sleep hygiene, and overall improved mental health [17,18]. On the other hand, a study conducted on a digital detox for smartphone users for 24 hours measured the effect on three parameters (mood, anxiety, and craving) on four different occasions. The results showed that only desire was affected, suggesting that a long period of smartphone usage might not be indicative of digital addiction [19]. Hence there seem to be varying results indicating the need for a personalized approach.

Tiffany Shlain [20], in her book '24/6: The power of unplugging one day a week', talks about how it's necessary to take a break from technology and smartphones once a day according to convenience to promote balance and recharge oneself with social interactions. She talks about putting aside a day for self-reflection and regaining control of one's life. The tips shared in her books are simple yet effective and are a means of promoting digital wellness.

## Discussion

Digital and online activity of students may include using the web and online services for social networking, education, information gathering, and entertainment. Adolescents, teenagers, and students need to analyze their use of various websites, mobile apps, electronic gadgets, and devices and differentiate between using digital technology for study and other purposes [12]. If the students' digital indulgence is more for activities other than academics, it may affect their progress and performance. Secondly, nowadays, if the quantum of online learning resource material is overwhelming in the education field, that too may adversely affect digital wellbeing.

To prevent the emergence of digital stress and its implications, it is vital to teach young students the effective use of digital and online resources. Some of the measures that may help to improve digital well-being are - capacity building of students for practical use and handling of digital and online resources [13], emphasizing active learning in the classroom and lab/fields, and promoting a healthy digital learning environment to facilitate digital creation, innovation, networking, and collaboration in academics and research [14], and use of interventions like digital detox and apps [15,17].

There has been sufficient data suggesting that using digital interventions promotes and helps an individual attain overall wellbeing. The use of digital detox programs, although proven effective, has drawbacks in the period and compliance of individuals [19]. Digital wellbeing apps have the advantage of easy-to-use technology and being accessible, proving to be the aptest link in reducing technological use. Using new and strategic app-based interventions has shown better progress and can cater to a much larger population. A positive association has been seen in using digital tools as they have demonstrated reduced stress and anxiety levels in a controlled setting [15].

Despite temporary and simple incorporable solutions that can easily be indulged in one's lifestyle, there have been profound difficulties in adhering to self-defined boundaries. It has been challenging to tweak habits and make lifestyle changes even when it is goal-oriented and is for the maintenance of well-being. Social settings leading to altered behavior and already developed habits and patterns pose a problem following the required regimens by customized apps and interventions. There is a constant need for motivation and reminders to keep focus and adhere to the targets. Since there is inconclusive evidence on the effectiveness of digital well-being due to various variables present, there is a prerequisite to personalizing digital well-being requirements by customizing an individual's specific needs. The limitations in using interventions for achieving digital well-being are seen to be a 'vantage point' in future studies and can help better understand this concept.

## Conclusions

The importance of digital wellness is to enable users to be more productive and engage in activities that promote holistic growth using technology to attain healthy and active lifestyles. Digital well-being can create significant advances in research with appropriate data backup. It can be integrated to promote awareness in medical students and teachers and encourage education about the struggles faced with social media connectivity, the risk of stress, and burnout. It can help foster healthy digital habits and promote the need for better interpersonal relationships with family and colleagues. There has, however, been minimal knowledge regarding it, causing a standby on its inquiry. The limitations set in achieving digital well-being can be overcome by more empirical research and customizing it to fulfill an individual’s purpose and goals.
